# Gleanings from Our Exchanges

**Published:** 1873-04-15

**Authors:** 


### Gleanings from Our Exchanges.

### EFFECTS OF PREGNANCY ON TUBERCULOSIS.

*The Clinic*, March 8th, 1873, contains a translation of an article on Tuberculosis of the female sexual organs, by Prof. H. Lebert (Breslau), the main points of which are summed up in the following conclusions:

1. Internal genital tuberculosis may be the chief localization of the disease in the female sex, or it may be only secondary in position; we possess no certain signs of genital phthisis, even when priimtive.

2. The disease described as tuberculosis of the collum uteri does not in reality exist, or at least it is extremely rare, and its acceptance depends upon a confusion with the cheesy epithelial debris of diseased glands about the orificium uteri.

3. The influence upon phthisis of pregnancy, and the puerperal bed, makes itself manifest most between the ages of 20–30, especially between, 25–30 and next between 30–40.

4. If phthisis be arrested in the young girl, it usually manifests itself upon marriage so soon as pregnancy occurs, both during pregnancy and during the puerperal bed, sometimes in the first, sometimes not until a later pregnancy.

5. In exceptional cases, the tuberculous patient may remain healthy in spite of repeated pregnancies and puerperia; in some of these cases, however, the children are feeble, and a certain percentage die early.

6. Advanced phthisis usually hinders conception; early phases of the disease do not interfere with it, and permit, usually, the full course of pregnancy.

7. Abortion, pregnancy, and the puerperal state, act injuriously, on the average, in three-fourths of cases, upon the development and rapid advance of phthisis.

8. The puerperal bed may not only excite the disease when the predisposition exists, but it acts relatively still worse; it accelerates the fatal termitation even more frequently than pregnancy. Exceptionally, however, a phthisis which has made rapid advances during pregnancy, takes on a slower and more favorable course in the puerperal bed.

9. Neither pregnancy nor the puerperal bed exercise any definite influence upon the localization or form of tuberculosis.

10. The bad influence of pregnancy and the puerperal bed manifests itself more especially where there is a hereditary predisposition, and the same hereditary favors; also, in marked degree, the outbreak of the disease during pregnancy.

11. Should phthisical women have a good getting-up period, they have, in the rule, but little milk, and can only exceptionally nourish their offspring. The children are mostly scrofulous, and, later on, tuberculous.

USE OF QUININE IN FEBRILE DISEASES OF CHILDREN.—E. Hagenbach (*Jahrb. fur Kinderheilk.*, March, 1872).—The author's object in this communication is to prove the favorable action of quinine in reducing the temperature in febrile diseases of children, by which means the disease is rendered a milder one, and in many cases complications of troubles of the nervous system and respiratory organs are prevented. He uses quinine in typhoid fever, and in all the various febrile diseases, when the temperature is continuously high for several days; he combines it with baths between 20° and 24° C., repeated every three hours as long as the temperature remains above 39° C. In pneumonia, he uses baths with older children; with smaller children wrapping in cold sheets is often preferred as less dangerous; at the same time he gives quinine. Scarlatina, the author treats almost exclusively with baths and quinine. This treatment has also been used with good effect in his later cases of erysipelas. Other diseases enumerated as so treated are acute rheumatism, acute periostitis, coxitis, with high fever, and several other surgical diseases. It is the combined action of the bath and of the quinine that the author lays stress upon. The quinine is given in one large dose, or in two smaller doses, at half-hour intervals, and when the temperature is below 39° C., it is left off. No bad after-effects were noted. The amount to be given varies with the age. For children between one and two years old, from five to sixteen grains; between three and five years, from eight to sixteen grains; between six and ten years, from ten to twenty grains; between eleven and fifteen years, from ten to thirty grains. The largest doses are generally to be preferred. The beneficial effects upon the sensorium, the circulation, subjective symptoms, etc., by baths, cold wet sheets and quinine, are, beyond doubt, as great in children as in adults, the strength is better kept up and convalescence hastened.—*Boston Med. & Surg. Journal.*

GALVANISM OF THE SYMPATHETIC IN GRAVES'S (BASEDOW'S) DISEASE.—Dr. M. Meyer (*Berliner Klinische Wochenschrift*, No. 39, 1872) records four cases of Graves's disease in females, where brilliant results were obtained by galvanizing the cervical sympathetic for a considerable time (during 20, 72, 60 and 84 sittings respectively in the four cases). A weak current was passed through the two sympathetics; then the one pole was applied to the submaxillary region, and the other to the shut eye, or to the goitre on the corresponding side; and the current passed for from two to three minutes. The effect of this treatment upon the dispersion of the exophthalmos and goitre is described as excellent—often after but a few sittings. The influence was also great upon the general health and upon the menstrual derangements, but less upon the palpitation.

It is remarkable that in all four cases the right side was especially affected, and that in one case there was no goitre.

Variola and Typhus Fever occurring at the same Time, in the same Individual, and Running their Course Together.—In the *Berliner Klin. Wochenschr.*, 1872, No. 11, Dr. Th. Simon states that an individual laboring under a recent slight attack of small-pox, was admitted as a patient into the Hamburg Hospital. Shortly after his reception there were developed in him the premonitory symptoms—intumescence of spleen, disturbance of sensorium, and the peculiar aspect of the stools—of fever, evidently of the typhus type. During the vesication of the pustules, there made its appearance a very copious eruption of roseola. The case terminated favorably.

Dr. Simon took opportunity to state that he had, during the prevalence of the variolous epidemic in Hamburg, frequently witnessed the early and wide-spread prevalence, among typhus patients, of a roseolar eruption, most probably the result of an influence exercised by the presence of the variolous epidemic.—*Centralblatt f. d. Med. Wissenschaften*, May 18, 1872, from *Berliner Klin. Wochenschr.*, 1872, No. 11.

---

Location for Sale.—On account of ill health a physician wishes to dispose of his property, situated in a growing village of 1,000 inhabitants, in Warren County, Ill., on the line of the Rockford, Rock Island & St. Louis R. R. The property consists of a handsome new residence, barn, and other buildings, and twenty-five acres of improved grounds. The owner has practiced medicine in the neighborhood for the past fourteen years, and is doing a business of from $3,000 to $4,000 a year. For terms and further particulars address "*Location*," care proprietors Medical Examiner, 792 Wabash ave.

